# Knowledge and Oral Health Attitudes among Parents of Children with Congenital Heart Disease

**DOI:** 10.5005/jp-journals-10005-1076

**Published:** 2011-04-15

**Authors:** Reshma Suvarna, Kavita Rai, Amitha M Hegde

**Affiliations:** 1 Postgraduate Student, Department of Pedodontics and Preventive Children Dentistry, AB Shetty Memorial Institute of Dental Sciences Deralakatte, Mangalore, Karnataka, India; 2 Professor, Department of Pedodontics and Preventive Children Dentistry, AB Shetty Memorial Institute of Dental Sciences Deralakatte, Mangalore, Karnataka, India; 3 Professor and Head, Department of Pedodontics and Preventive Children Dentistry, AB Shetty Memorial Institute of Dental Sciences Deralakatte, Mangalore, Karnataka, India

**Keywords:** Oral health, Attitudes, Knowledge, Congenital heart disease.

## Abstract

Congenital heart disease (CHD) is a devastating complex of diseases resulting from defects of development. It affects more than 1 of every 100 live births. Early preventive dental care should be adjusted to the special needs of these children in their first years of life. Knowledge of parental attitudes and experiences of dental care are therefore important.

**Aim :** This study was done to assess the knowledge and attitudes among parents of children with congenital heart disease towards oral health and dental care.

**Materials and methods :** Parents (n = 105) of children with congenital heart disease of an age ranging from 0 to 16 years were included in the study. A questionnaire was used to assess the knowledge and oral health attitudes.

**Results :** The parents’ knowledge was fair but the oral health attitudes were not very satisfactory. The parents in this study also recognized the importance of oral health for the well-being of rest of the body.

**Conclusion :** The results of this study indicate that parents’ and children’s attitudes toward oral health and dental care need to be improved.

## INTRODUCTION

Congenital heart disease (CHD), abnormalities in the structural development of the heart, occurs in approximately 8:1000 live births. With improved detection, diagnosis, and progress of surgical and anesthetical methods, the number of surviving children is increasing.^1^ The epidemiology of heart disease in children has changed over the past 3 to 4 decades. The increased survival rate of children with congenital heart disease has increased and the overall decrease in rheumatic valvular heart disease has been observed in the developed countries. CHD now constitutes the predominant underlying condition for infective endocarditis (IE) in children over the age of two years in these countries.^2^ The causative microorganisms for infective endocarditis in more than 60% of the patients with positive hemoculture are streptococci, with viridians streptococci representing 50% of this group, especially *Streptococcus sanguis, S. mitior* and *S. mutans.* Poor oral hygiene may give rise to frequent bacteremia under normal physiological conditions, leading to a permanent risk of developing the disease.

Children with cardiac crisis have significantly poorer dental health practices than the healthy group.^1,3-5^ There are several reasons for this, such as chronic intake of sugared medicines, increased tooth susceptibility from developing enamel defects, greater consumption of sweets in compensation, negligence of oral hygiene as a result of a greater concern with cardiac disease.^5^

Early dental health problems are common in children with severe CHD, and there are complicated background factors often associated with nutrition, medication, and the demanding situation of their families. Lower frequencies of regular dental care have been displayed in children with CHD than in those without this medical problem. It is an essential challenge for pediatric dentists to provide these children, whose medical health can be hazardous as a result of poor dental health, a dental service that is adjusted to their needs. Knowledge of parental attitudes and experiences of dental care plays a key role in providing an early preventive dental care to the special needs of children with CHD in their first years of life.^1^

## MATERIALS AND METHODS

The subjects comprising the population of this study, i.e. the parents of children with congenital heart disease were recruited from a leading cardiac center in Bengaluru. The parental awareness and attitude was assessed through a questionnaire. A total of 95 subjects were included in the study whose children were between infancy to 16 years of age. The follow-up of 95 subjects who participated in the study, of which 86 completed questionnaires, were evaluated and formed as part of our study. The parental study group comprised of 67(63.8%) females and 38(36.2%) males.

The clearance for the study was obtained from the head of the pediatric cardiology department. Since there were a significant number of patients who hailed from outside Karnataka and who were non-Kannada speaking, questionnaires were also printed in Hindi and English to prevent any language barrier.

All the subjects were requested to complete the questionnaire. The questionnaire included fifteen items which were split into four different categories covering oral hygiene habits, awareness of gingival health and plaque, knowledge and awareness of dental and general health, and attitudes toward professional dental care. Assessments of oral hygiene habits included items on oral hygiene methods used, brushing intervals, and role of parents’ supervision of it.

## RESULTS

Following the data analysis, it was found that 87.4% of the children used brush and paste to maintain oral hygiene whereas 5.7% of them used mouth wash and a meager 2.3% used tooth picks. The remaining 4.6% followed other methods. Around 56.3% of them brushed the teeth twice daily and the remaining (42.5%) brushed once a day. About 1.2% of the single time brushers reported of brushing only before bed time (Table 1).

Regarding their awareness on gingival health 41.37% of them said they had no knowledge about it and 39.08% said that redness of gingiva could be prevented by brushing and flossing. About 12.64% of them said it could be prevented by eating soft food and 6.89% thought that it could be prevented with vitamin C consumption. Their knowledge on plaque was also found to be very unsatisfactory where a large percentage (62.06%) did not know what it meant and 14.94% said it was yellow discoloration of tooth whereas 10.34% said it was hard permanent deposits on the tooth, only 12.64% of them knew that it was soft deposits on the tooth (Table 2).

Regarding the awareness and knowledge of dental and general health, when asked about the effect of sweets on dental health, 85.9% replied in affirmative and 14% replied with a negative answer. Another 2.35% said that they were not aware of the effects of sweets on dental health. In reference to the effect of discolored teeth on their child’s appearance 85.9% replied that it does have an effect, 8% said that it does not have any effect and 7% replied that they did not have any knowledge about it. Around 82% of the subjects in the study group were aware of the significance of oral health on the cardiac status, whereas 8% in the same study group did not think so and 9% of the subjects had no clue about any of the general health issues. Though 88% of the parents did understand that treatment of toothache is as important as any organ in the body, 4% disagreed to this and 8% of the population in the study group was ignorant about it (Table 3).

**Table 1:** **Table 1:** Oral hygiene habits among the study population

		*Frequency*		*Percentage*	
*Oral hygiene methods used:*					
• Toothbrush and paste		76		87.4	
• Mouthwash		5		5.7	
• Toothpicks		2		2.3	
• Others (mango and neem leaf, charcoal, etc.)		4		4.6	
*Brushing intervals:*					
• At morning		37		42.52	
• Before bed		1		1.15	
• Before bed and at morning		49		56.3	
• Other times		0			
*Role of parents in supervision of oral hygiene:*					
• Parents watch and advise		72		82.75	
• Parents only advice but do not watch		7		8.05	
• Parents never cared		8		9.19	

**Table 2:** **Table 2:** Awareness of gingival health and plaque

		*Frequency*		*Percentage*	
*What does plaque mean?*					
• Soft deposits on teeth		11		12.64	
• Heavy deposits on teeth		9		10.34	
• Tooth discoloration		13		14.94	
• Do not know		54		62.06	
*How to prevent redness of gingiva?*					
• Brushing and flossing		34		39.08	
• Soft food		11		12.64	
• Vitamin C		6		6.89	
• Do not know		36		41.37	

**Table 3:** **Table 3:** Knowledge and awareness of dental and general health

		*Frequency*		*Percentage*	
*Do sweets affect dental health?*					
• Yes		73		85.9	
• No				12	
14					
• Do not know		2		2.35	
*Do discolored teeth affect your*					
*child’s appearance?*					
• Yes		73		85.9	
• No		7		8	
• Do not know		6		7	
*Does the health of mouth and*					
*teeth impact the health of body?*					
• Yes		70		82.4	
• No		7		8	
• Do not know		8		9.4	
*Does treatment of toothache as*					
*important as any organ in the body?*					
• Yes		74		87.1	
• No		3		3.5	
• Do not know		7		8.23	

On assessment regarding the frequency of dental visits, 78% said that they visited when only in pain, 17% occasionally and only 2% visited regularly. Concerning the necessity of visiting the dentist regularly, 64% knew that regular visits to the dentist are very much important, 16% did not agree with this thought, and 20% replied that they did not know anything about this (Table 4).

Remainder of the questionnaire revealed that when the parents were asked whether poor oral health jeopardized the health of the heart, 67% replied positively, 20% did not comply, and 7% said that it does not matter at all. Also, when asked whether they were interested in educating themselves about the importance of oral health and its implication on general well-being, 82% replied that they were interested, whereas 5% replied that they were not interested at all, and 2% replied negatively.

## DISCUSSION

Previous studies on knowledge, attitudes and health practices have shown that the cardiac group had significantly poorer dental health practices than the healthy group.^1,3-5^ Even their guardians’ knowledge was not satisfactory with regards to the importance of the maintenance of good oral health for prevention of infective endocarditis. In a recent study of children with CHD in northern Sweden, it is suggested that children with severe CHD should receive dental care in clinics for pediatric dentistry, particularly at early ages.^1^

**Table 4:** **Table 4:** Attitudes toward professional dental care

		*Frequency*		*Percentage*	
*How often do you visit the dentist?*					
• Regularly		2		2.4	
• When in pain		66		77.6	
• Occasionally or never		17		20	
*Are regular visits to the dentist*					
*necessary?*					
• Yes		54		63.5	
• No		14		16.5	
• Do not know		17		20	
*Reasons behind not visiting/dislike*					
*visiting:*					
• Fear		12		15.4	
• High cost		22		26.7	
• No clinic nearby		12		15.4	
• No time		5		7.6	
• No specific reason		35		42.3	
*Driving factor for your last visit:*					
• Toothache		52		61	
• Parents advice		8		10.6	
• Dentists advice		9		12.5	
• Other reasons		4		4.7	
*Are you aware that poor oral*					
*health is not good for the heart?*					
• Yes		57		67.1	
• No		17		20	
• Does not matter at all		6		7.1	
*Are you interested in educating* *yourself about the importance of* *oral health, its implication on* *general well-being (especially* *the heart)?*					
• Yes		75		88.2	
• No		2		2.4	
• Not interested		4		4.7	

This survey shows that a high percentage of children do brush the teeth twice or once a day but this chore was not always assisted by the parents. Also, the intervals of brushing were found to be irregular. These findings are concurrent with the study done by Al-Omiri and colleagues in North Jordan.^6^

Attentiveness of gingival health and plaque were also found to be unsatisfactory where most of them were not even aware of the idea of plaque. Most of them thought it to be discoloration of tooth, some thought it to be heavy deposits whereas some did have the impression that it was soft deposits on the tooth. Similar findings were observed in the study done on children from North Jordan.^6^ Knowledge of the prevention of redness of gingiva was also very deficient. Only one third of the study group knew it could be prevented by brushing and flossing and the rest thought it could be prevented by consumption of soft food and vitamin C.

Perception of dental and general health was only acceptable. Where bulk of the subjects did show adequate knowledge regarding the effect of sweets on the dental health, they also agreed with the fact that treatment of toothache was as important as any organ in the body. Also, the realization that health of mouth and teeth do impact the health of the body was prevalent among a good number of subjects in this study.

Majority of the study subjects often visited the dentist when only in pain, a very small percentage visited only occasionally and the number of subjects who visited regularly were almost nil. These findings are contrast or rather surprising in the sense that a good number of the same study groups were aware of the fact that regular visits to the dentist are necessary. These findings are concurrent with the previous studies,^1,3,4^ where the reasons for poor motivation to visit the dentist for preventive treatment and routine dental care in spite of ample awareness were due to the lack of parental encouragement and motivation to visit the dentist regularly, which could be reflected in the attitudes of the children. The reasons for the lack of attendance at the dental clinics were not very specific, cost factor being the second most common reason followed by fear. This is in difference with the previous study where fear was the most prominent factor for not visiting the dentist.^6^ The driving factor for the last visit to the dental clinic among the participants was largely for toothache followed by dentists’ and parents’ advice. This could be accredited to the deficient awareness regarding the preventive approaches and the lower compliance towards dental treatment.

Realization that poor oral health jeopardizes health of the heart was prevalent in majority of the subjects but this knowledge is not being implemented toward actions like increasing the visits to dental clinics and benefiting from the preventive treatment. A small portion of the study group should be motivated even to seek information and then only preventive program will be effective. The interest in educating themselves about the importance of oral health and its implication on the general well-being (especially the heart) was widespread among the subject, but the essential motivation to channelize the knowledge into effective deeds is what we have to aim at in the first place to see any improvement in this regard.

There is considerable awareness regarding the importance of oral health in these children, not much effort is put into implementing this knowledge into action. Also, the general oral hygiene habits were only satisfactory. Parent’s knowledge and attitudes about the importance of oral health care and their fears about dental treatment influenced their children’s dental care. Therefore, parent’s and children’s attitudes toward oral health and dental care need to be improved. Comprehensive oral health educational programs for both children and their parents are required to achieve this goal.

## RECOMMENDATIONS

1. Parents should be thoroughly educated regarding the importance of maintaining oral hygiene in these children:
  - As there are an increasing number of children with severe congenital heart disease, it is important to develop a dental care that is adjusted to these children’s needs.
  - Parental help should be given to these children during tooth brushing.
  - Dental surgeons should attend cardiac assessment clinics to ensure that each child has the benefit of a full dental diagnosis and access to primary preventive care.
2. Children should receive dental care in clinics for pediatric dentistry, particularly at early ages.
3. At the earliest an individual treatment plan to maintain oral health, based on risk assessment, should be established for all these children with cardiac disease.
